# Biomimetic Mineral Synthesis by Nanopatterned Supramolecular-Block Copolymer Templates

**DOI:** 10.1021/acs.nanolett.3c00480

**Published:** 2023-05-04

**Authors:** Susrut Akkineni, Gregory S Doerk, Chenyang Shi, Biao Jin, Shuai Zhang, Stefan Habelitz, James J De Yoreo

**Affiliations:** † Department of Materials Science and Engineering, University of Washington, Seattle, Washington 98195, United States; ‡ Physical Sciences Division, Physical and Computational Sciences Directorate, Pacific Northwest National Laboratory, Richland, Washington 99352, United States; § Center for Functional Nanomaterials, Brookhaven National Laboratory, 735 Brookhaven Avenue, Upton, New York 11973, United States; ∥ Department of Preventive and Restorative Dental Sciences, School of Dentistry, University of California, San Francisco, California 94143, United States

**Keywords:** block copolymers, nanopatterning, calcium phosphate, amelogenin, biomineralization

## Abstract

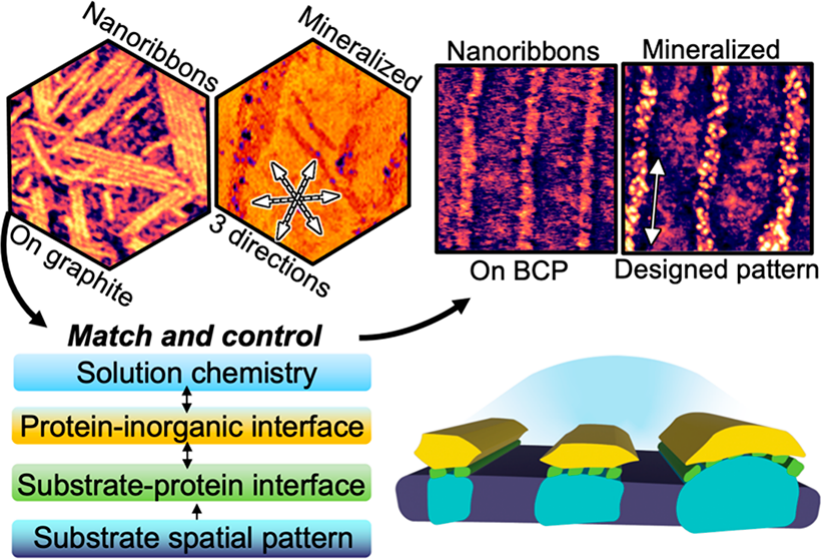

Supramolecular structures of matrix proteins in mineralizing tissues are known to direct the crystallization of inorganic materials. Here we demonstrate how such structures can be synthetically directed into predetermined patterns for which functionality is maintained. The study employs block copolymer lamellar patterns with alternating hydrophilic and hydrophobic regions to direct the assembly of amelogenin-derived peptide nanoribbons that template calcium phosphate nucleation by creating a low-energy interface. Results show that the patterned nanoribbons retain their β-sheet structure and function and direct the formation of filamentous and plate-shaped calcium phosphate with high fidelity, where the phase, amorphous or crystalline, depends on the choice of mineral precursor and the fidelity depends on peptide sequence. The common ability of supramolecular systems to assemble on surfaces with appropriate chemistry combined with the tendency of many templates to mineralize multiple inorganic materials implies this approach defines a general platform for bottom-up-patterning of hybrid organic–inorganic materials.

Supramolecular assemblies of biopolymers, in the form of nanofibers, nanosheets, nanotubes, or nanoribbons, can be potent templates for bottom-up synthesis of inorganic nanomaterials,^1−3^ both in bulk solution^4^ and when patterned on surfaces.^5,6^ While numerous surface-based studies have related the thermodynamic, kinetic, stereochemical, and spatial constraints at organic templates deposited on surfaces to their ability to direct inorganic crystallization,^7−14^ they were largely applied to simple, nonhierarchical structures, typically comprising self-assembled monolayers (SAMs) of small molecules. In contrast, similar analyses using supramolecular assemblies with secondary, tertiary, or quaternary structures have been rare,^5,6,15^ despite the need for such information to define design rules for biomolecular templates and the availability of methods for such analyses, even in more complex systems. Complementing investigations of mineralization by organic templates, several studies have demonstrated that supramolecular assemblies of peptides, proteins, and peptoids can be patterned on atomically flat materials such as graphene, highly oriented pyrolytic graphite (HOPG), MoS_2_, WS_2_, WSe_2_, BN, MoSe_2_, or mica by directing self-assembly into islands of continuous linear arrays (lamellae) along crystallographic directions of the underlying substrate.^6,16−19^ These two complementary advances offer an opportunity to develop a robust synthetic platform for the more sophisticated patterns of hybrid organic–inorganic materials generally required for biomedical or technological applications. To explore this concept, here, we investigate three fundamental questions. First, can we pattern model protein or peptide assemblies on patterned surfaces and subsequently use them to direct mineralization with high fidelity? Second, can we template mineral filaments and platelets that mimic those seen in biominerals by using this approach? Third, what are the underlying physicochemical design rules for template mineralization?

To answer the above questions, we direct mineralization of calcium phosphate using self-assembling nanoribbons (NRs) of amelogenin (Amel) peptides associated with tooth enamel formation as the supramolecular template and lamellar block copolymer (BCP) films as the platform for patterning the template. We perform these studies for a range of pattern dimensions using two prototypical Amel peptide sequences that are mineralized from both an aqueous solution of precursor ions and a polymer-induced liquid-like precursor (PILP) of calcium phosphate. Our results demonstrate that patterning of supramolecular self-assembly and subsequent mineralization into filaments and platelets can be achieved, provided that the appropriate template designs, mineral precursors, and methods are used. In situ atomic force microscopy (AFM) shows that the supramolecular protein structures are oriented as designed and offer high-fidelity patterning of mineralization. This is further confirmed spectroscopically through photo-induced force microscopy (PiFM), which shows that the peptides assembled with the correct conformation selectively on one of the two BCP regions (polystyrene (PS)). These results demonstrate that mineralization-directing supramolecular assemblies that typically self-assemble on atomically flat surfaces can also be assembled into predefined patterns that can direct mineral formation with high fidelity for potential applications in tissue or device engineering.

In our experimental design, peptide analogues of phosphorylated amelogenin (pAmel) NRs were selected as a model supramolecular template due to their higher mineral nucleation rates compared to their nonphosphorylated versions, well-characterized interfacial properties, and robust self-assembly protocol.^6,20^ pAmel NRs—p14P2 (GHPGYINF p(S) YEVLT) and p14P2Cterm (GHPGYINF p(S) YEVLT DKTKREEVD)—self-assemble into amphiphilic NRs with β-sheet conformation at pH 1.94 in bulk solution that can then be patterned on HOPG via hydrophobic and van der Waals interactions or, alternatively, self-assembled from monomers directly on HOPG.^6^ Both sequences are potent nucleators of amorphous calcium phosphate (ACP) producing high nucleation rates (kinetics) due to low nucleation barriers (i.e., low surface energy).^6^ For both sequences, as for many biomineral systems,^21−23^ the initial formation of the amorphous phases is followed by transformation into a final crystalline phase, which, in the case of pAmel NRs, is apatite. Of the two sequences, p14P2Cterm includes a segment of amino acids (DKTKREEVD, labeled Cterm) appended at the C terminus,^6^ which is known to exhibit high affinity for binding to apatite.^24^ The peptide has a higher net charge, induces higher ACP nucleation and growth rates on HOPG, and exhibits a faster phase transformation from ACP to apatite.^6^ The Cterm segment was also predicted to be more flexible and have no β-sheet conformation in solution and on HOPG compared to the rest of the sequence.^24^

To pattern pAmel NRs, we employed a versatile substrate, silicon-supported thin films of self-assembled block copolymer (BCP) lamellae with alternating hydrophilic and hydrophobic stripes, which were previously shown to pattern self-assembly of Fibrinogen,^25^ Sbpa S-layer proteins,^26^ and collagen fibers.^26^ BCP thin films offer excellent chemical contrast, with stripe widths that can be easily tuned to allow the exploration of lateral region sizes and confinement at the nanoscale.^27,28^ Additionally, BCPs can be tuned to assemble into three-dimensional (3D) scaffolds or exploited for 3D printing.^29,30^ Consequently, they provide a platform for engineering hybrid nanomaterials and approaches to designing or exploring synthetic systems for the synthesis of bone and tooth enamel.^31^ First, we used a blend of a simple BCP, polystyrene-*block*-poly(methyl methacrylate) (PS-*b*-PMMA), with PS and PMMA homopolymer, which formed lamellar patterns consisting of 50.3 ± 4.1 nm wide PS stripes with 170.9 ± 5.2 nm periodicity on the surface and less than 1 nm difference in height between PS and PMMA stripes (Figure 1), labeled as **50 nm PS**. Later, we adjusted the blend composition to produce patterns with PS stripes of width 95 nm (labeled as **95 nm PS**) and 150 nm (labeled as **150 nm PS**) to study the impact of increasing lateral region size on crystallization. For details on the synthesis of BCP patterns, see Methods and Table 1 in Supporting Information.

**Figure 1:**
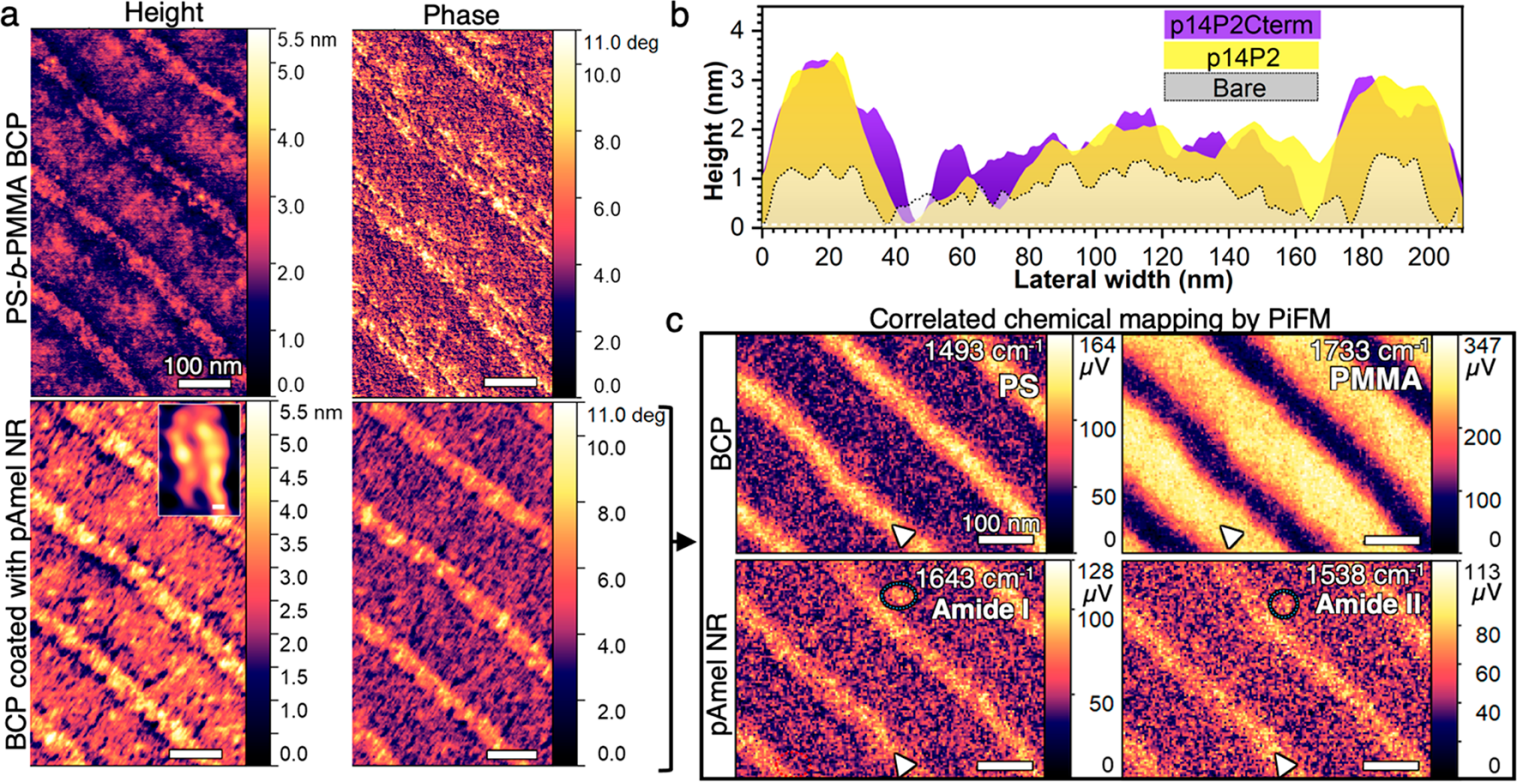
Representative in situ AFM and PiFM images of pAmel NRs assembled on 50 nm PS stripes of PS-*b*-PMMA BCPs. (**a**) BCP before and after incubation of 0.05 mg/mL p14P2 for 30 min shows height of PS and PMMA stripes increase by ∼1.4 and ∼0.6 nm, respectively, along with significant change in phase contrast of PS stripes. Inset image shows morphology of two NRs on PS (scale bar: 10 nm). (**b**) Comparison of representative height profiles drawn perpendicular to stripes on bare BCP and p14P2-coated BCPs shown in (A) and p14P2Cterm-coated BCP. (**c**) PiFM surface map of p14P2-coated BCP (dried) at excitation wavelengths specific to BCP and β-sheet pAmel NR shows pAmel NRs assembled on PS stripes (bright stripes). Arrows point to excited regions. Circles highlight the cross-β-sheet pAmel NR on PMMA.

In this design, the hydrophobic and aromatic PS are expected to bind the aromatic amino acid-rich residues of the pAmel peptides via hydrophobic and van der Waals interactions to induce β-sheet NR self-assembly directly onto the PS substrate from peptides in solution. This is facilitated by using a freshly prepared peptide solution at acidic (pH 1.94) and low concentration (0.05 mg/mL) conditions in which NRs are rare when deposited on a variety of substrates (HOPG, mica, MoS_2_). Moreover, the hydrophilic PMMA is both stable at pH 1.94 needed for protein assembly and charge neutral at pH 7.4 used for mineralization. PMMA stripes were also designed to be wider than the PS stripes to minimize coalescence of mineral particles growing on adjacent PS stripes and enable straightforward interpretation via AFM. Thus, as a stringent test of protein and mineral patterning, we investigated whether pAmel NRs retain their functionality and bias nucleation of ACP on PS stripes, which, without further functionalization, remain inert during mineralization.

In situ AFM imaging of the change in morphology, height, and phase contrast between the PMMA and PS stripes upon exposure to solutions of pAmel peptides indicates that pAmel NRs assemble on the PS stripes (Figure 1a). The average thicknesses of p14P2 and p14P2Cterm NRs on PS were 1.4 ± 0.3 and 1.5 ± 0.2 nm, respectively (Figure 1b. For the procedure used to calculate thickness, see Methods in Supporting Information). The NR length ranged from 10 to 150 nm, forming one or two rows of NRs on 50 nm wide PS stripes (inset to Figure 1a). Both the thickness and minimum length for both sequences correlate with those of β-sheet NRs (or protofibrils) obtained in our previous study.^6^ However, we note that NRs longer than 150 nm, which are typically observed on HOPG, were not observed on PS stripes here. We hypothesize that the van der Waals interactions between peptide monomers and/or NRs and aromatic carbon of PS necessary for diffusion, aggregation, and growth of long NRs are weakened here by the higher surface roughness of PS stripes (*R*_average_ = 0.26 ± 0.03 nm, *R*_maximum_ = 1.32 ± 0.26 nm), combined with the upright orientation and/or relatively disordered nature of aromatic carbon headgroups in PS, in comparison to aromatic carbon with recumbent orientation and crystalline arrangement on atomically flat surfaces of HOPG.^6,32,33^

In contrast to their assembly on PS, β-sheet NRs do not appear to form on the PMMA regions. Overall, the PMMA region is rougher after exposure to the pAmel solution, with the center of the PMMA stripe having an average height about 0.6 nm greater relative to the edge, but whether this reflects the adsorption of a disordered layer of monomeric peptides is unclear. We also observed ∼3 nm tall particles sparsely distributed on the PMMA stripes; these may be small fragments of thicker cross β-sheet aggregates of pAmel (bilayer β-sheets with hydrophilic surfaces on both faces), which form in the bulk solution and deposit on the surface.^6,20^

To confirm the above assessment of the topographical changes, PiFM was performed to obtain correlative nanoscale chemical imaging of the different species and protein conformations on the substrates (after removing excess peptide solution and drying the BCP surfaces). Excitation wavelengths specific to PS (1493 cm^–1^), PMMA (1733 cm^–1^), and β-sheet conformation of Amel NR (Amide I: 1643 cm^–1^ and Amide II: 1538 cm^–1^) were obtained from previous AFM-infrared measurements.^34,35^ The PiFM images, shown for BCP-p14P2 NRs (Figure 1c), highlight the locations of the chemical species at their respective excitation wavelengths (brighter stripes indicated by white arrows). The results indeed correlate with the in situ AFM measurements and clearly demonstrate PS stripes predominantly bind pAmel NR with a β-sheet conformation, while the ∼3 nm tall particles on PMMA (circled) could be cross-β-sheet NRs, whereas the proposed random-coil peptide layer on PMMA could not be mapped with high contrast due to low response and thickness below the PiFM detection limit. However, the relatively low signal in the Amide I and II maps from the PMMA stripes as compared to those of the PS regions shows the lack of β-sheet NRs and that any peptides on those regions remain monomeric or random coil. As a result, we obtain a heterogeneous interface consisting of a periodic array of amphiphilic/hydrophilic PMMA and hydrophilic pAmel NR stripes.

To test whether the patterned NRs can nucleate minerals, pAmel NRs were assembled on BCP surfaces in an incubator at 37 °C (100% relative humidity) for at least 60 min, and the peptide solution was thoroughly exchanged with pure water. Subsequently, nucleation experiments were performed on these substrates with calcium-phosphate solutions supersaturated with respect to ACP (σ_ACP_) for which no precipitation occurred in the bulk solution.^5,6,15^ To observe and quantify the mineral nucleation events, we used in situ AFM with a flow-through apparatus to generate constant composition environments (Figure 2a–c), thus obtaining quantitative information about the nucleation dynamics, kinetics, and patterning fidelity over time. Alternatively, the substrates were mineralized by immersion in supersaturated solutions for more than 20 min, washed with water, and dried for analysis (Table of Contents figure). Post mineralization, the nucleated particles were carefully extracted from the surface (Methods in Supporting Information) and characterized by high-resolution transmission electron microscopy (HRTEM) and selected area electron diffraction (SAED) (Figure 2d), confirming the formation of ACP.

**Figure 2:**
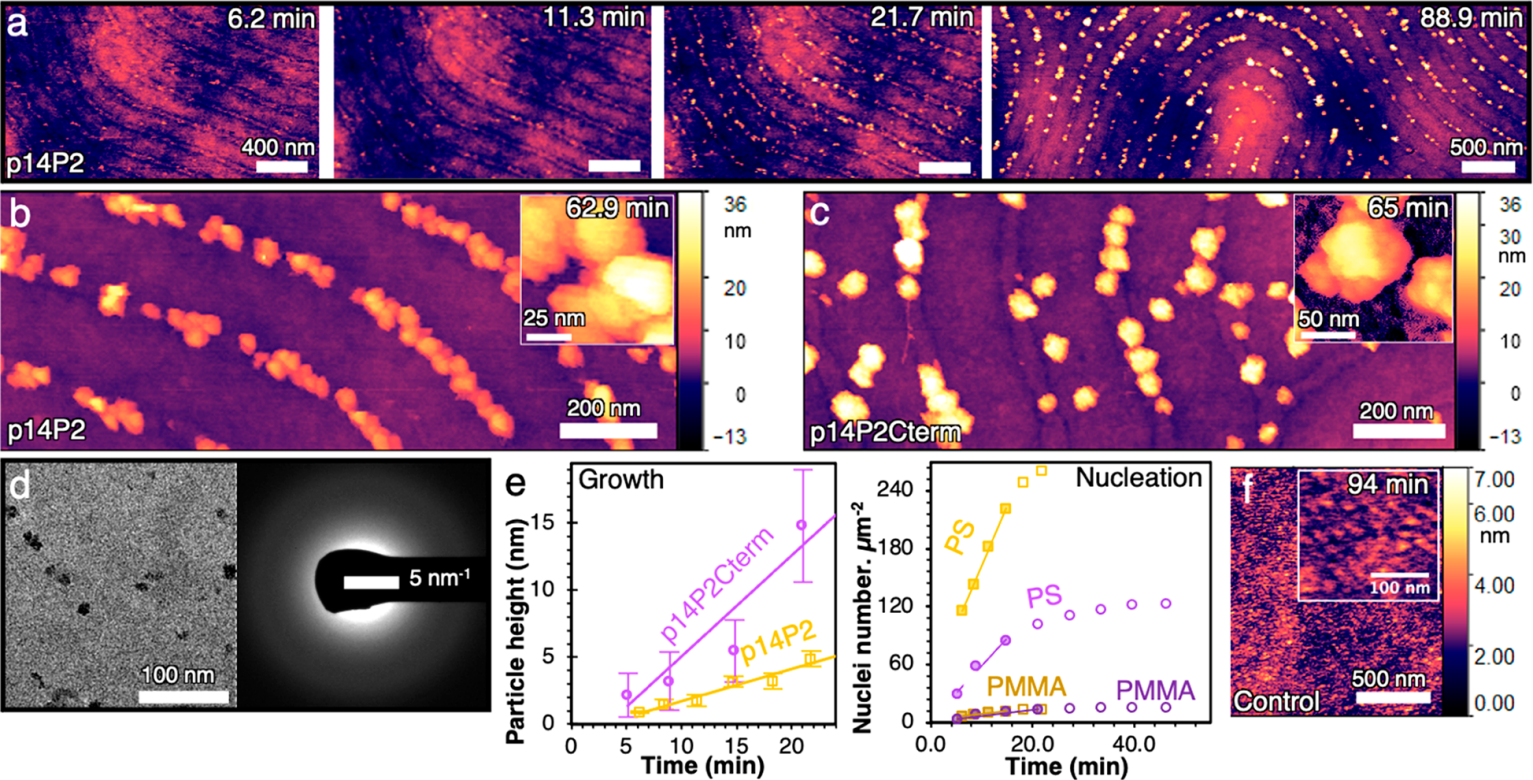
pAmel NR-PS stripes nucleate ACP under a constant chemical potential. In situ AFM shows BCPs coated with (**a**) p14P2 nucleates and grow calcium phosphate particles over time. *t* = 0 min is defined as the time when solution is introduced into the flow cell. (**b**) p14P2 has high fidelity, and (**c**) p14P2Cterm has lower fidelity and larger particles. (**d**) Under TEM and SAED, particles were amorphous. (**e**) Comparison of representative ACP nucleation and growth rates for p14P2 (yellow) and p14P2Cterm (violet). For nucleation rates, the number of nuclei on PS and PMMA are separated and normalized to their corresponding area (Methods in Supporting Information), and the error is standard deviation. (**f**) Absence of mineralization on BCP (control with 12 nm wide PS stripes and 24 nm periodicity) without pAmel NR coating even after 94 min.

At σ_ACP_ = 0.04, we found that NRs of both sequences exhibited a high ACP nucleation density on the PS stripes (Figure 2a–d). In fact, the nucleation rates measured for NRs assembled on PS were even greater than those obtained previously on HOPG^6^ (12.8× higher for p14P2 NRs and 1.9× higher for p14P2Cterm), though the relative magnitude for the two sequences was reversed from that observed on HOPG where p14P2Cterm was the more potent nucleator (Table 2 in Supporting Information). Specifically, we find that nucleation and growth rates on NRs assembled on PS were 2.2× higher and 3.2× lower, respectively, for p14P2 than for p14P2Cterm NRs (Figure 2e). The ACP nucleation rate on PS-p14P2 was also ∼20 times higher than on the PMMA regions of both the PMMA-p14P2 and PMMA-p14P2Cterm patterns. The minor occurrence of nuclei on the PMMA regions may be attributable to the sparsely dispersed cross-β-sheet NRs described above or possibly defects in the PMMA layer. The morphology of ACP particles on NRs on PS were similar to those on HOPG from our previous study.

Of the two sequences, p14P2 displayed higher fidelity of mineral patterning than did p14P2Cterm. Further analysis of the in situ AFM data shows this is due to the higher nucleation rate on PS-p14P2. Moreover, due to the difference in growth rates, many of the ACP nuclei on PMMA-p14P2 dissolved overtime, whereas the ACP on PMMA-p14P2Cterm grew in size, leading to a loss in patterning fidelity.

In stark contrast to the high rates of ACP nucleation observed for the NRs on PS, no ACP nucleation was seen on the two negative controls, bare BCPs with 50 and 12 nm PS stripe widths (Figure 2f). These findings demonstrate that the templating potency of pAmel NR-solution interfaces observed on HOPG is transferable to the PS stripes and that patterns of functional supramolecular templates can be generated with our protocol.

Previous work showed that Amel NRs exposed to solutions of calcium phosphate-based polymer-induced liquid precursor (PILP) induce formation of ACP that evolves into single-crystal apatite filaments growing along the NRs,^35^ thus reproducing the filamentous growth habit of apatite crystals comprising tooth enamel.^36^ PILP, which is produced by adding high molecular weight poly(aspartic acid) (pAsp) or other polycationic polymers and proteins to calcium carbonate or phosphate solutions, has been implicated as the mineral precursor phase in natural biomineral systems,^37,38^ including those based on apatite.^39,40^ Therefore, to test whether patterned pAmel NR-PS interfaces can template filament-like apatite from a PILP phase, we exposed them to calcium phosphate PILP solutions made with 27 kDa poly-Aspartic acid (pAsp) as the biopolymer additive.^35,40^ Additionally, we varied the PS stripe width and spacing to determine the impact of increasing pAmel NR-PS surface area and, thus, the number density of potential nucleation sites and separation as well as the versatility of these templates. (The altered BCPs had 95.2 ± 23.3 nm wide PS stripes at a 174.5 ± 12.4 nm center-to-center distance (labeled as 95 nm PS) and 151.7 ± 14.6 nm wide PS stripes at a 249.7 ± 21.8 center-to-center distance (labeled as 150 nm PS)).

Multiple in situ AFM experiments with both pAmel NR sequences demonstrated that all PS stripe widths do indeed template formation of smooth apatite crystals from PILP solution. p14P2Cterm NRs typically produced micron long filament-shaped crystals, while the longest crystal observed on p14P2 NRs was 171 nm. In contrast, bare BCP films produced no filament or plate-shaped crystals on the PS regions. Thus, further studies with other values of the PS stripe width were performed with p14P2Cterm (Figure 3a–c) due to our interest in filament-shaped apatite. The results show that the width of the apatite crystals directly depends on the width of the PS stripes (i.e., the lateral NR or potential nucleation site number density) in the order 150 ≥ 95 > 50 nm PS (Figure 3d) and is therefore easily tunable. The average thicknesses and widths of these crystals were 20.1 ± 3.6 and 78.9 ± 10.8 nm for 50 nm PS, 7.8 ± 1.8 and 101.3 ± 18.7 nm for 95 nm PS, and 36.4 ± 27 and 108.7 ± 31.3 nm for 150 nm PS, respectively. Each plate-shaped mineral is a single crystal, exhibiting the *ab* planes ((202), (211), and (222)) of apatite (Figure 3e) similar to apatite filaments in enamel.^36^ This may also be true for the filament-shaped mineral in Figure 3a,b, but it could not be clearly verified due to extensive aggregation.

**Figure 3:**
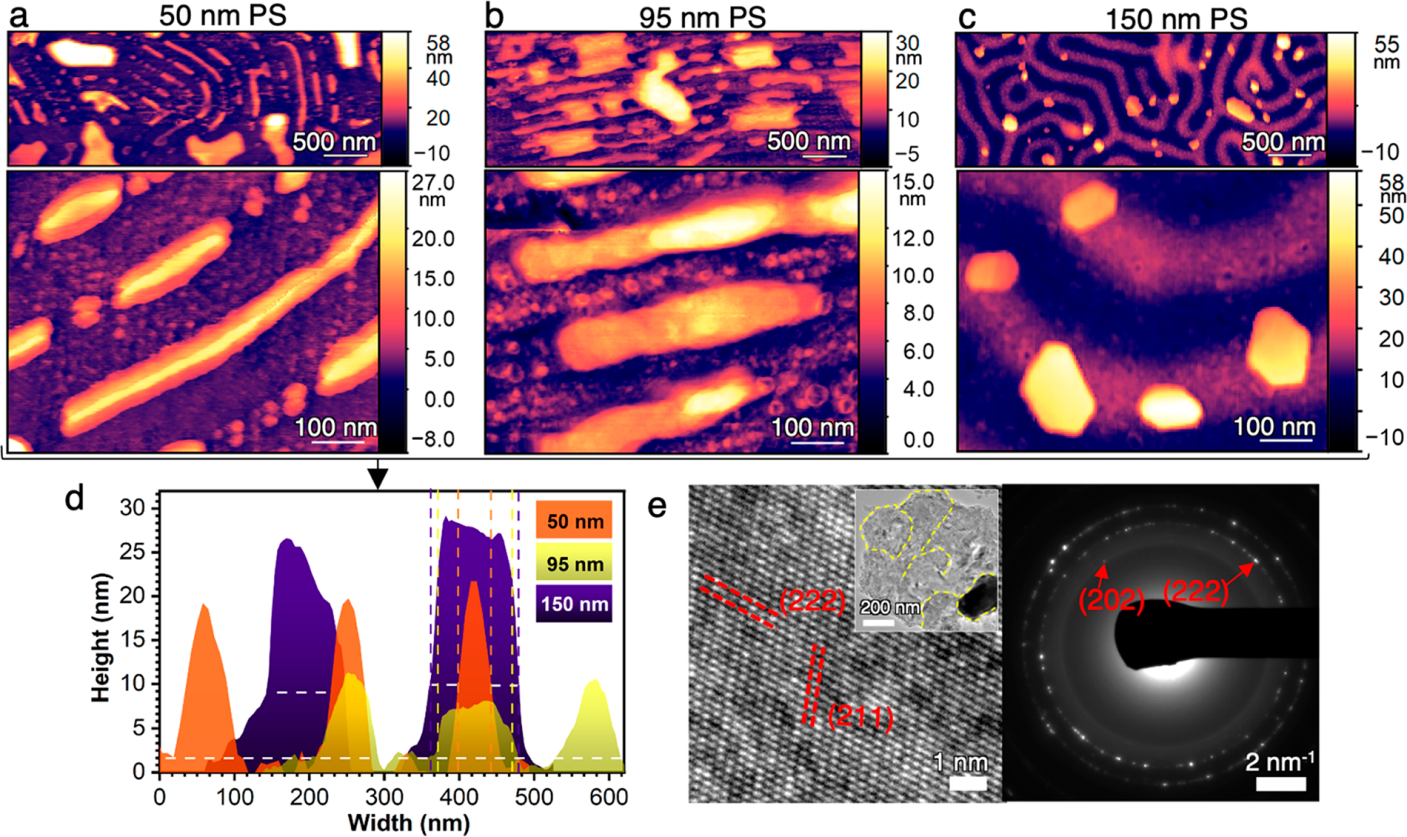
Smooth apatite crystals with tunable dimensions are obtained using PILP. In situ AFM shows filament-like morphology of the mineral particles with low surface roughness formed on p14P2Cterm-PS stripes for (**a**) 50 nm, (**b**) 95 nm, and (**c**) 150 nm wide PS stripes. (a) was imaged in PILP solution, and (b) and (c) were imaged in water after mineralization by PILP. Note that the high-magnification image in (a) was captured with a double tip; see Supporting Information Figure S1 for a low-resolution single-tip image used for measuring the height profile. (**d**) Comparison of height profiles of crystals formed on 50, 95, and 150 nm p14P2Cterm-PS stripes; white horizontal dashes indicate base of the particle (PS stripe), and color-coded vertical dashes indicate the filament width. (**e**) Representative HRTEM and SAED analysis of extracted particles (shown for crystals in c) confirm the particles are crystalline with lattice and reflections specific to apatite; Inset: low-magnification TEM image and corresponding SEAD pattern showing the aggregated single crystals and their grain boundaries (yellow dashed line).

Despite the above results, we observed a few limitations with the PILP-based method that need to be addressed in future efforts. First, the total area of the patterns covered by mineral (i.e., the yield) was lower than that obtained through nucleation of ACP from a polymer-free solution (Figure. 2). Second, the transformation of ACP to apatite could not be followed by AFM to provide a clear mechanistic understanding, despite attempting various environmental conditions described in the literature (heating to 50 °C,^35^ cooling to 10 °C,^13^ continuous perfusion of PILP, drying,^13^ and suspending inverted substrates on PILP solution^13^). To overcome these challenges and ensure high reproducibility, we will systematically explore varying PILP compositions and delivery methods in the future.

Besides pAmel NRs, other proteins that can be assembled at solid–liquid interfaces, such as collagen and SbpA S-layer proteins, which both self-assemble on mica, have also been shown to assemble on BCP films. Thus, in principle, BCPs can serve as a general platform for patterning of supramolecular structures that are known to assemble at solid–liquid interfaces. In addition, given that individual supramolecular assemblies have been shown to template multiple mineral phases (for example, collagen, which is a natural scaffold for calcium phosphate nucleation, also directs formation of iron oxide, calcium carbonate, and silica),^41^ BCP-directed assembly of supramolecular templates may represent a highly generalizable platform for patterning mineralization. To effectively employ this strategy, the following physicochemical constraints can be powerful tools for investigation and translation. These are informed by the study presented here and our previous analyses on pAmel NRs assembled on HOPG.^6^

First, the surface-binding interface of the supramolecular template needs to match one of the BCP chemistries to enable directed self-assembly. In addition, the assembled supramolecular template should be oriented such that the mineral nucleating interface is sterically unhindered by the precursors of the mineral in bulk solution. Neglecting these constraints leads to ineffective mineral nucleation, as seen on PMMA.

Second, for heterogeneous in vitro mineral nucleation on patterned polymeric surfaces (Figure 2), the mineral nucleation density must be high and growth rates must be low to get high surface coverage with high fidelity. This can be achieved by using low supersaturations (close to zero) and increasing the mineralization duration if larger particles are required; otherwise, the initial nuclei grow rapidly, block free nucleation sites, and exhibit a morphology dominated by the natural crystal growth habit.

Third, the difference in ACP nucleation rates between p14P2 and p14P2Cterm on HOPG versus PS suggests that supramolecular structures lacking flexible random-coil segments are more effective mineral templates on polymeric surfaces such as PS. We infer this based on observations of random-coil behavior in Cterm segment (DKTKREEVD) of NRs on HOPG and in solution.^6^ Here, the flexible Cterm likely complexes with mineral ions while inhibiting new nucleation along the β-sheet-templating region of the NR. A previous study also suggests the hydrophilic C terminus of amelogenin is effective in binding to apatite surfaces but appears to reduce the ability of amelogenin to nucleate mineral.^35,42^

To summarize, our study shows that the β-sheet conformation and interfacial properties of pAmel NRs, previously seen on HOPG,^6^ are conserved on the PS regions of the PS-*b*-PMMA BCP patterns. The pAmel NRs, especially p14P2 NRs, on PS can effectively confine the nucleation of ACP formed from supersaturated solutions. Both the number density and size of the mineral particles can be easily tuned through changes in the mineralization duration or peptide sequence. In contrast, disordered peptide monomers adsorbed on PMMA and uncoated PS and PMMAs surfaces are unable to nucleate and grow mineral. Furthermore, we find that the patterned NRs can template smooth filaments or platelets of apatite crystals from PILP solutions synthesized using acidic macromolecules, thus inducing an outcome that both matches that obtained with NRs of full-length amelogenin in bulk PILP solution and emulates the hypothesized in vivo function of pAmel NRs.^35,42^

From the findings reported here, we conclude that the bottom-up biomimetic synthesis of nanopatterned inorganic materials may be possible through spatially controlled nucleation sites generated by supramolecular structures. Typically, these structures have low interfacial energies with respect to the mineral (on atomically flat materials^6^) and display high mineral nucleation rates at low supersaturations. The ability to tune each parameter during systems design represents an exciting step toward scalable synthesis of sophisticated inorganic two-dimensional (2D) or 3D nanostructures for applications in electronics, medicine, energy, or catalysis.

## Supporting Information Available

Figure S1, Low-resolution single tip image of Figure 3a. Methods and materials for: pAmel NR solutions, PILP solutions, BCP substrates, substrates for mineralization. PiFM measurements, In situ AFM measurements and analysis, Table 1 with BCP compositions, Table 2 with ACP nucleation and growth rates, TEM measurements (PDF)
