# Supplementary material for: Biomimetic Mineral Synthesis by Nanopatterned Supramolecular-Block Copolymer Templates
Source: Nano Lett. 2023 May 4;23(10):4290–7. doi: 10.1021/acs.nanolett.3c00480 (PMC10215289; doi:10.1021/acs.nanolett.3c00480)
Supplement: Supplementary file 1 — nl3c00480_si_001.pdf [file nl3c00480_si_001.pdf]

Biomimetic mineral synthesis by nanopatterned supramolecular-block copolymer templates

Susrut Akkineni[#‡], Gregory S Doerk[∂], Chenyang Shi[‡], Biao Jin[‡], Shuai Zhang[#‡], Stefan Habelitz[ǁ] and James J De Yoreo[#‡*]*

[#] Department of Materials Science and Engineering, University of Washington, Seattle, WA 98195, USA.

[‡] Physical Sciences Division, Physical and Computational Sciences Directorate, Pacific Northwest National Laboratory, Richland, Washington 99352, USA.

[∂] Center for Functional Nanomaterials, Brookhaven National Laboratory, 735 Brookhaven Avenue, Upton, NY 11973, USA.

[ǁ] Department of Preventative and Restorative Dental Sciences, School of Dentistry, University of California, San Francisco, CA 94143, USA.

Corresponding Authors Emails

Stefan Habelitz[ǁ] : stefan.habelitz@ucsf.edu

James J De Yoreo[#‡] : james.deyoreo@pnnl.gov

Supporting Information

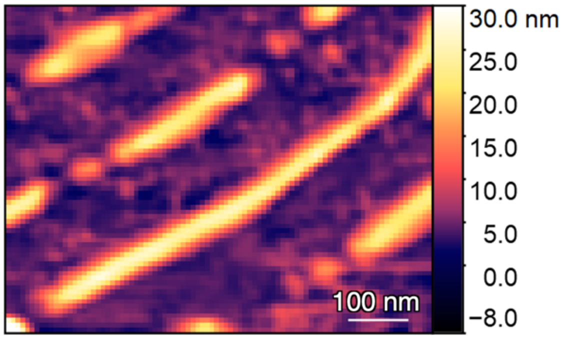

Figure S1. Low resolution in situ AFM image of Figure 3a, mineral filaments on p14P2Cterm coated 50 nm PS BCPs, captured with single tip.

Methods and Materials

pAmel NR solutions

Previously established protocol for pAmel NR self-assembly was used (1). Briefly, the lyophilized peptide from Elim Biopharmaceuticals, Inc. (CA, USA) was dissolved in 10 mM HCl pH 1.94 (37 wt.% HCl in H_2O, 99.999% trace metals basis, Sigma-Aldrich, diluted with ultrapure deionized water purified by MilliQ Elix Essential 3 and Advantage A10). Solutions were vortexed and sonicated for 15 minutes in a Branson ultrasonic bath (M5800H, 40 kHz frequency) until a clear solution is obtained. The stock solutions were 1 mg/mL in concentration, i.e., molarities of 596 µM for p14P2 and 360 µM for p14P2Cterm, and immediately diluted to 0.05 mg/mL using 10 mM HCl (pH 1.94) then used for assembly on BCPs.

PILP solutions

PILP solutions of calcium phosphate were prepared following established protocols (2) and used according to our previous publication (3), described as follows. Poly-L-aspartic acid sodium salt, MW=27 kDa (pAsp) was purchased from Alamanda Polymers, Inc (AL, USA) and 99.99% pure $CaCl_2$, KH_2PO_4 and NaCl were purchased from Sigma-Aldrich. Stock solution for each reagent was independently prepared by dissolution in 25 mM pH 7.4 Hepes buffer, concentrations were 0.8 mg/mL pAsp, 36 mM $CaCl_2$, 4.2 mM KH_2PO_4 and 600 mM NaCl. The solutions were stored at 4 °C when not in use. The final PILP solution was freshly prepared by sequentially mixing 1:1 volumes of stock solution in the following order: pAsp to $CaCl_2$, then NaCl to pAsp and $CaCl_2$, and finally KH_2PO_4 to the mixture of pAsp, $CaCl_2$ and NaCl to make up the final diluted concentrations of 0.1 mg/mL pAsp, 4.5 mM $CaCl_2$, 150 mM NaCl and 2.1 mM KH_2PO_4.

BCP substrates

Immediately prior to peptide assembly or mineralization, the patterned BCP surface was cleaned by thoroughly rinsing with more than 25 mL of ultrapure deionized water, then immediately dried with clean N_2 gas. If the patterns were exposed to ambient atmosphere for long durations, the adsorbed organic contaminants on the surface were removed by briefly rinsing with 1% solution of Alconox detergent, or alternatively with 200 μL of 10 mM HCl (solvent for peptides) repeated three times, prior to rinsing with

water and drying. The BCP samples were fabricated using established materials and methods described below.

Materials: A hydroxyl-terminated polystyrene-*random*-poly(methyl methacrylate) (PS-*r*-PMMA-OH) random copolymer "brush" [60 mol % styrene, determined by ^{13}C NMR, M_n = 9.2 kg/mol, PDI = 1.35 (determined by gel permeation chromatography relative to PS standards)] was provided by the Dow chemical Company already dissolved in propylene glycol monomethyl ether acetate (PGMEA) and diluted to a concentration of 1% (w/w) in PGMEA. Various lamellae-forming polystyrene-*block*-poly(methyl methacrylate) (PS-*b*-PMMA) diblock copolymers were purchased from Polymer Source and used without further purification. These were 36.5 kg/mol PS-*b*-PMMA (M_n = 18.5−18 kg mol^{-1}; PDI = 1.05), 51 kg/mol PS-b-PMMA (M_n = 25−26 kg mol^{-1}; PDI = 1.09), and 1051 kg/mol PS-*b*-PMMA (M_n = 536−515 kg mol^{-1}; PDI = 1.1). Likewise, PS (M_n = 3.5 kg/mol, PDI = 1.05) and PMMA (M_n = 3 kg/mol, PDI = 1.14) homopolymers were purchased from Polymer Source and used as received. All neat and blended polymer solutions were prepared using PGMEA solvent at a concentration of 2% (w/w). PGMEA, tetrahydrofuran (THF), and acetone were purchased from Sigma-Aldrich. Test-grade (100) oriented silicon wafers purchased from University Wafers were used as substrates for polymer film deposition and treatment.

BCP film preparation**:** Substrates were initially cleaned by oxygen plasma treatment (March Plasma CS1701F, 100 mTorr, 20 W, 60 s). The PS-*r*-PMMA-OH brush was then grafted to the substrate as described previously (4) to provide a "neutral" surface with balanced surface energies between PS and PMMA that helps ensure vertical orientation of the lamellar domains. Briefly, this is accomplished by spin-coating the PS-*r*-PMMA-OH solution onto samples at 1500 rpm, baking the samples for 5 minutes at 252 °C in a nitrogen-enriched environment to promote the brush grafting to the substrate, and rinsing in PGMEA at 3000 rpm to remove ungrafted polymer brush. Polymer blend solutions were prepared by mixing different polymer solutions (all 2% by weight in PGMEA) in prescribed weight ratios to achieve the desired polymer mass ratios. Binary blends of diblock copolymers or ternary PS-*b*-PMMA/PS/PMMA blends were then spin-cast at 1500 rpm to achieve films with thicknesses ranging from 60 to 90 nm. Self-assembly was achieved by thermal or solvent vapor annealing (SVA). Samples with varying linewidths referred to in the main text were obtained from the ternary block copolymer/homopolymer blends by adjusting the blend composition or solvent vapor annealing protocol; the salient preparation details corresponding to each pattern designation used in the main text are provided in Table 1.

Table 1: Polymer Film Compositions and Annealing Protocols

Designation	Film Composition (weight %)	Annealing Protocol (Nitrogen Flow Rate, SCCM)
12 nm PS	36.5 kg mol^{-1} PS-*b*-PMMA/51 kg mol^{-1} PS-*b*-PMMA (75/25)	Thermal annealing (N/A)
50 nm PS	1051 kg mol^{-1} PS-*b*-PMMA/ 3 kg mol^{-1} PMMA (40/60)	THF SVA (0.4)
95 nm PS	1051 kg mol^{-1} PS-*b*-PMMA/ 3.5 kg mol^{-1} PS/ 3 kg mol^{-1} PMMA (40/20/40)	THF SVA (0.4)
150 nm PS	1051 kg mol^{-1} PS-*b*-PMMA/ 3.5 kg mol^{-1} PS/ 3 kg mol^{-1} PMMA (40/30/30)	Acetone SVA (No flow)

BCP film annealing: Thermal annealing was performed by baking samples for 5 minutes at 252 °C in a nitrogen-enriched environment. Solvent vapor annealing was performed as described previously (5) by placing samples in a chamber with a controlled vapor pressure of either THF or acetone. Briefly, the annealing chamber consisted of a canister machined from a single aluminum block with a central pedestal to hold samples above an annular solvent reservoir. A Peltier plate is used to maintain a constant temperature of 21.0 ± 0.2 °C during annealing. Annealing is commenced by placing a sample on the pedestal, adding 4-5 mL of solvent to the reservoir, and covering the chamber with a 6.5 mm thick borosilicate glass

window. Nitrogen purging at flow rates from 0 or 0.4 standard cubic centimeters per minute (SCCM) was used to control the maximum polymer film swelling, which was monitored *in situ* by measuring the film thickness at 20 s intervals during annealing using spectral reflectance in the visible range (Filmetrics F20-UV). Minimum polymer volume fractions (given by the ratio of initial to maximum solvent swollen film thickness) during solvent vapor annealing were in the range of 0.28 to 0.3 for THF, and 0.33 for acetone. After 1 hour, annealing was rapidly quenched by removing the glass cover, drying within ~1 s. After solvent vapor annealing, samples were baked for 30-60 s at 252 °C in air.

Substrates for mineralization

BCP samples without a peptide coating were used directly after cleaning. For peptide coated BCPs, 20-100 μL of freshly made 0.05 mg/mL peptide solution was drop-cast on a cleaned BCP surface and incubated at 37 ºC in a sealed chamber (Relative Humidity ~100%) for 1 hour following our previous protocol for HOPG. After incubation, solutions on HOPG were gently exchanged with 1 mM HCl (pH 3.1) first then H_2O, three times each, to remove unbound protein/peptide without disassembly.

For mineralization without AFM (ToC figure) described in main text, the BCP samples were incubated in 0.05 mg/mL of the peptide solution for 1 hours at 37 ºC in sealed tube. The substrates were then immersed in 1 mM HCl (pH 3.1) first then H_2O, three times each for 2 seconds, to remove unbound protein/peptide without disassembly. Subsequently, 1.5 mM $CaCl_2$ and 14.9 mM KH_2PO_4 (pH 7.4) were independently prepared, mixed in a tube and the BCP substrates were incubated in the mixture for at least 20 minutes for nucleation and growth to take place. Following this, the samples were immersed in water to remove the excess mineral solution and dried in vacuum for AFM and TEM analysis.

Photoinduced Force Microscopy (PiFM)

BCP samples without a peptide coating were used directly after cleaning. For peptide coated BCPs, the substrates for mineralization described above were used for the PiFM after removing the excess water and allowing the wet peptide film to dry slowly in vacuum. All samples were characterized in air using a VistaScope PiFM (Molecular Vista Inc.) coupled to a Laser Tune QCL with a wave number resolution of 0.5 cm^{-1} and a tuning range from 800-1800 cm^{-1}. The microscope was operated in dynamic mode with HQ:NSC15/Cr-Au probes (MikroMasch). The data was processed in Surface Works software (Molecular Vista Inc.).

***In situ* Atomic Force Microscopy (AFM)**

Silicon nitride cantilevers with Si tip (Bruker SNL-10, spring constant k: 0.12 N/m or 0.24 N/m) treated with UV/ozone for 15 minutes were used for all experiments. Peptide self-assembly and ACP nucleation were performed using Bruker MultiMode 8 AFM, operated in liquid using tapping mode at room temperature (25˚C). PILP experiments were performed on the Cypher VRS AFM (Asylum Research) with a Peltier heater/cooler stage to adjust sample temperature and operated in liquid. Gwyddion and ImageJ/Fiji software were used for offline processing of images, feature size measurements and counting nuclei. Detailed methodologies are available in a previous publication (1).

<u>NR self-assembly</u>: The bare BCP surface was first imaged in 10 mM HCl (pH 1.94) to ensure absence of contaminants, then 200 μL of freshly made 0.05 mg/mL peptide solution was injected into the AFM liquid cell and imaged immediately.

<u>Measurements of peptide layer thickness:</u> Line profiles (more than 10 for each type) with line width of 3 pixels were drawn perpendicular to each stripe, e.g., Figure 1b. Average thickness of peptide layers (NRs or monomers) on PS or PMMA is obtained from the difference of the average height of the stripe between coated and uncoated samples. To obtain height values of individual stripes, the boundary between PS and PMMA stripes was used as a baseline. The boundary appears as a valley in Figure 1b (white dashed line) or as the darkest regions in height images of Figure 1a. Some regions of the boundary remain uncoated with peptide when exposed to peptide solutions, therefore, suitable to obtain thickness from the peak value.

Nucleation and growth experiments: All BCP samples were characterized in pure water before flowing the mineral precursor solutions. The protocol and analyses for mineralization experiments at constant composition (supersaturation, σ_{ACP} = 0.04) are identical to procedures used for amelogenin nanoribbon coated HOPG in a previous publication (1). Briefly, 1.5 mM $CaCl_2$ and 14.9 mM KH_2PO_4 (pH 7.4) were independently prepared and filtered three times with a cellulose acetate filter (pore size of 0.1 μm) before immediate use. The filtered $CaCl_2$ and KH_2PO_4 solutions were independently and continuously pumped at 37.25 μl/min and combined at the inlet of the AFM liquid cell using a custom T-junction.

Calculation of nucleation and growth rates: The nucleation and growth rates are extracted from AFM images using established methods (1). To determine nucleation rate specifically on PS or PMMA stripes shown in Table 2, the number of nuclei on PS and PMMA stripes were tracked and counted separately and normalized with the total area of their corresponding regions (PS or PMMA) in the entire image.

Table 2. Nucleation (J_0) and growth rates (V) under supersaturated calcium and phosphate solutions (σ_{ACP} = 0.04) on peptide coated 50 nm PS BCPs (Figure 2) and HOPG (Akkineni et al.).

**Sequence**	**Nucleation rate (J_0 in nuclei μm^{-2} min^{-1})**			**Growth rate (V in nm/min)**
	**PS**	**PMMA**	**HOPG (1)**	
p14P2	12.434	0.603	0.974	0.239
p14P2Cterm	5.623	0.625	2.986	0.755

PILP-based mineralization: All BCP substrates were first characterized in pure water then exposed to the PILP solution. In this method, the AFM Peltier cooler/heater stage temperature was set to 25 ˚C. After the sample temperature equilibrated, the cantilever holder was disassembled and the water on the sample was rapidly exchanged with the PILP solution twice without dehydration of the peptide film on the surface. The PILP solution was incubated on the substrate for 15 min, then the water on the cantilever holder was replaced with 25 μL of PILP solution to avoid dilution. Immediately after this step, the holder is rapidly brought in contact with the liquid on the BCP and close to the surface (to avoid drying at the edges due to necking/meniscus) and imaged. Experiments at 10 ˚C were performed similarly but by changing the stage temperature, which produced results identical to those shown in main text (Figure 3c).

Transmission Electron Microscopy (TEM)

Sample preparation: Immediately after mineralization, the substrate was rinsed with 200 μL of ultrapure water to quench the reaction and remove the mineral precursor solution and unbound particles. The substrate was then immediately dried with N_2 gas. The mineral particles were extracted from the surface by using a pipette to drop-cast 5-10 μL of ethanol on the BCP surfaces and withdrawing the liquid after 30 seconds. The ethanol causes the BCP film to swell (characterized by AFM), which release the surface-bound mineral particles into the withdrawn solution. The ethanol and mineral mixture are then immediately drop-cast onto lacey carbon grids (Ted Pella, USA) and the solution is left to incubate on the grid for 5 min before removing the excess and allowing the grids to dry in ambient air before storage in vacuum until characterization. To verify whether there was any change in crystallinity during extraction, we prepared multiple samples, mineralized them for different durations of time, and verified the morphology by AFM and then by TEM, following a procedure developed and used previously for analysis of calcium phosphate phases nucleated on surfaces (1, 6, 7).

High-resolution TEM (HRTEM) and Selected Area Electron Diffraction (SAED): All grids were imaged with a field emission Titan ETEM 80–300 kV (Thermo Fisher Scientific) operated at 300 kV. Detailed characterization of the ACP and apatite particles is described elsewhere (1, 6). Briefly, the FFT (Fast Fourier Transform) of HRTEM on several particles characterized as ACP lacked lattice fringes corresponding to crystalline calcium phosphate and SAED shows a broad diffraction band where d-spacing

for crystalline calcium phosphate is expected (8). In contrast, for particles characterized as apatite in PILP experiments, the FFT of HRTEM images and SAED showed the lattice fringes and sharp bands at d-spacings, respectively, for apatite.

References

1. S. Akkineni, *et al*., Amyloid-like amelogenin nanoribbons template mineralization via a low-energy interface of ion binding sites. *Proc Natl Acad Sci U S A* **119**, e2106965119 (2022).
2. F. Nudelman, *et al*., The role of collagen in bone apatite formation in the presence of hydroxyapatite nucleation inhibitors. *Nat Mater* **9**, 1004–1009 (2010).
3. Y. Bai, *et al*., Protein nanoribbons template enamel mineralization. *Proc Natl Acad Sci U S A* **117**, 19201–19208 (2020).
4. G. S. Doerk, K. G. Yager, Rapid Ordering in "Wet Brush" Block Copolymer/Homopolymer Ternary Blends. *ACS Nano* **11**, 12326–12336 (2017).
5. G. S. Doerk, R. Li, M. Fukuto, K. G. Yager, Wet Brush Homopolymers as "Smart Solvents" for Rapid, Large Period Block Copolymer Thin Film Self-Assembly. *Macromolecules* **53**, 1098–1113 (2020).
6. W. J. E. M. Habraken, *et al*., Ion-association complexes unite classical and non-classical theories for the biomimetic nucleation of calcium phosphate. *Nat Commun* **4**, 1507 (2013).
7. J. Tao, *et al*., Control of Calcium Phosphate Nucleation and Transformation through Interactions of Enamelin and Amelogenin Exhibits the "goldilocks Effect." *Cryst Growth Des* **18**, 7391–7400 (2018).
8. J. Tao, H. Pan, Y. Zeng, R. Xu, R. Tang, Roles of amorphous calcium phosphate and biological additives in the assembly of hydroxyapatite nanoparticles. *Journal of Physical Chemistry B* **111**, 13410–13418 (2007).
